# Application of the World Health Organization Quality of Life Instrument, Short Form (WHOQOL-BREF) to patients with cataract

**DOI:** 10.4178/epih.e2016005

**Published:** 2016-02-04

**Authors:** Ali Gholami, Mahmood Tavakoli Araghi, Fatemeh Shamsabadi, Mahdiye Bayat, Fatemeh Dabirkhani, Farhad Moradpour, Kamyar Mansori, Yousef Moradi, Abdolhalim Rajabi

**Affiliations:** 1 Department of Public Health, School of Public Health, Neyshabur University of Medical Sciences, Neyshabur, Iran; 2 Department of Epidemiology, School of Public Health, Iran University of Medical Sciences, Tehran, Iran; 3 Student Research Committee, Neyshabur University of Medical Sciences, Neyshabur, Iran; 4 Social Determinants of Health Research Center, Kurdistan University of Medical Sciences, Sanandaj, Iran; 5 Department of Epidemiology, School of Public Health, Shahid Beheshti University of Medical Sciences, Tehran, Iran; 6 Pars Advanced and Minimally Invasive Manners Research Center, Pars Hospital, Iran University of Medical Sciences, Tehran, Iran

**Keywords:** Cataract, Quality of life, World Health Organization, Reproducibility of results, Questionnaires, Cross-sectional study, Iran

## Abstract

**OBJECTIVES:**

Cataract is a prevalent disease in the elderly, and negatively influences patients’ quality of life. This study was conducted to study the application of the World Health Organization Quality of Life Instrument, Short Form (WHOQOL-BREF) to patients with cataract.

**METHODS:**

In this cross-sectional study, 300 patients with cataract were studied in Neyshabur, Iran from July to October 2014. The Iranian version of the WHOQOL-BREF questionnaire was used to measure their quality of life. Cronbach’s alpha coefficient, Pearson’s correlation coefficient, the paired t-test, the independent t-test, and a linear regression model were used to analyze the data in SPSS version 16.0 (SPSS Inc., Chicago, IL, USA).

**RESULTS:**

The mean age of the participants was 68.11±11.98 years, and most were female (53%). The overall observed Cronbach’s alpha coefficient for the WHOQOL-BREF was 0.889, ranging from 0.714 to 0.810 in its four domains. The total mean score of the respondents on the WHOQOL-BREF was 13.19. The highest and lowest mean scores were observed in the social relationship domain (14.11) and the physical health domain (12.29), respectively. A backward multiple linear regression model found that duration of disease and marital status were associated with total WHOQOL scores, while age, duration of disease, marital status, and income level were associated with domains one through four, respectively (p<0.05).

**CONCLUSIONS:**

The reliability analysis conducted in this study indicated that the WHOQOL-BREF scale exhibited an acceptable degree of internal consistency in the measurement of the quality of life of patients with cataract. It was also found that the patients with cataract who were surveyed reported a relatively moderate quality of life.

## INTRODUCTION

Cataract is a common disease in the elderly, and is especially prevalent in those older than 65 years. Cataract manifests as double or distorted vision, halo or glare vision, blurry vision, colors appearing differently, and gradual deterioration of the vision [1]. Surgical and non-surgical management are two strategies for treating cataract. The non-surgical strategy is typically performed during the early stages of cataract development, while the operative strategy is generally performed when a cataract affects patients’ daily activities [2]. Snellen visual acuity has been widely used in clinical practice to measure a patient’s visual function. Vision problems due to cataract have negative effects on patients’ quality of life. Quality of life is defined by World Health Organization (WHO) as “an individual’s perception of their position in life in the context of the culture and value systems in which they live and in relation to their goals, expectations, standards and concerns” [3]. The simple evaluation of the prevalence of cataract and its associated medical problems cannot convey the full meaning of its impact on the physical, mental, and social well-being of affected individuals. Quality of life measures allow for a more comprehensive understanding of the problems due to cataract. However, in order to study quality of life, we must be able to measure it. The World Health Organization Quality of Life Instrument, Short Form (WHOQOL-BREF) questionnaire is a commonly utilized generic measure of quality of life that is used to measure quality of life in healthy people and in different groups of patients [4-20]. The WHOQOL-BREF questionnaire is available in many languages, and has been translated into Persian and validated in Iran by Nedjat [21]. Therefore, this study was conducted to study the utility of the WHOQOL-BREF in assessing the quality of life of patients with cataract.

## MATERIALS AND METHODS

In this cross-sectional study, data were collected between July and October 2014 from all cataractous patients who received surgical treatment at two hospitals in the city of Neyshabur (northeastern Iran). All patients provided informed consent after being acquainted with the purpose of the study.

### Procedure and study instrument

In this study, the questionnaires were filled out in personal interviews, but before the interviews, all participants were informed that their responses would remain confidential. The validated Persian-language version of the WHOQOL-BREF was used in this study. This questionnaire contains two items assessing overall quality of life and general health, as well as 24 other items divided into four domains: physical health (domain 1) with seven items, psychological health (domain 2) with six items, social relationships (domain 3) with three items, and environmental health (domain 4) with eight items. Each item is rated on a five-point Likert scale and scored from one to five on a response scale. According to the guidelines, the raw domain scores for the WHOQOL-BREF were transformed to a score between four and 20 [22]. The scores of each domain are scaled in a positive direction (i.e., lower scores denote lower quality of life). The mean score of the items in each domain is used to calculate the domain scores, which are ultimately transformed linearly to a scale of zero to 100 [23,24]. The inclusion criteria applied in this study were: (1) the presence of cataract, (2) residence in Neyshabur, and (3) agreement to participate in the study.

### Dependent and independent variables

In this study, the four domains of the WHOQOL-BREF questionnaire were considered dependent variables and other data (age, sex, education level, marital status, monthly income level, place of residence, and duration of disease) were considered independent variables. The age of the participants was represented by two categories: 65 years of age or younger and greater than 65 years of age. The education level of participants was classified as illiterate or literate. Marital status was categorized into two categories: single/divorced and married. Income level was divided into the two categories of ≤$170 and >$170 per month. The criterion of place of residence was categorized as urban and rural. The time interval from the detection of cataract until the time of the survey was divided into two categories: ≤ 30 days and >30 days.

### Statistical analyses

In this study, the data were analyzed using SPSS version 16.0 (SPSS Inc., Chicago, IL, USA). The descriptive analyses included frequencies, percentages, ranges, means, and standard deviations (SD). The reliability of the WHOQOL-BREF domains and overall quality of life were assessed using Cronbach’s alpha, with scores of 0.70 and over deemed acceptable [25]. We used Pearson’s correlation coefficient to determine the level of agreement between the four domains of the WHOQOL-BREF. The paired t-test was used to compare the mean scores of the different domains of the WHOQOL-BREF. The independent t-test and a linear regression model were used to investigate the relationship between patients’ quality of life and their characteristics. Transformed scores were used for statistical analyses in all domains, and the level of significance was set at p<0.05 for all analyses.

## RESULTS

A total of 300 patients with cataract filled out the WHOQOL BREF questionnaire in this study. The characteristics of the study population are shown in Table 1. The mean age of the study population was 68.11±11.98 years. Of the participants in this study, 158 (53%) were female and 142 (47%) were male. Table 2 presents the missing responses, mean score, SD, and floor and ceiling effects for each item. The highest and lowest mean scores were observed in the personal relationship (3.99) and leisure activity (2.09) items, respectively. The percentage of respondents scoring at the highest level (ceiling effect) ranged from 2.3% to 35.3%, while the percentage of respondents scoring at the lowest level (floor effect) ranged from 1% to 32.3%. Cronbach’s alpha coefficient was applied to examine the internal consistency of the WHOQOL BREF scale (24 items) as well as its four domains. The Cronbach’s alpha coefficient of the WHOQOL BREF was adequate (0.889) for all 24 questions, with the following values for each domain: domain 1, 0.810; domain 2, 0.765; domain 3, 0.731; and domain 4, 0.714. As Table 3 shows, statistically significant correlations were found between each domain of the WHOQOL-BREF. Additionally, statistically significant correlations were observed between the overall quality of life item (Q1) and the scores of the various domains (Table 3). The paired t-test was used to compare the mean scores of the four domains of the WHOQOL-BREF. As Table 4 shows, significant differences were found between all domains of the WHOQOL-BREF (except for the difference between domain 2 and domain 4). The total mean score of the WHOQOL-BREF was 13.19 (65.95%). Among the different domains of the WHOQOL-BREF, the lowest and highest mean scores and percentages of responses indicating satisfaction were found for domain 1 (mean, 12.29, 51.95%) and domain 3 (mean, 14.11, 63.20%), respectively (Table 5). The mean score of the four domains and the total score of the WHOQOL-BREF according to the independent variables (sex, age, education level, marital status, income, duration of disease, and place of residence) are displayed in Table 5. As seen in Table 5, the means and percentages of responses indicating satisfaction in the total score and in domains 1, 2, 3 were lower in males than females, but this pattern was reversed in domain 4. Additionally, the means and percentages of responses indicating satisfaction were higher in all domains in married persons than in single persons (Table 5). As Table 5 illustrates, differences were found between different statuses regarding certain variables (age, sex, education level, marital status, monthly income level, place of residence, and duration of disease) in total and in the four domains of the WHOQOL at the level of significance of p<0.2. Table 6 displays the results of reverse multiple linear regression; it is clear that duration of disease and marital status were significantly associated with the total WHOQOL score. Age was associated with domain 1, duration of disease was associated with domain 2, marital status was associated with domain 3, and income was associated with domain 4.

## DISCUSSION

In this study, we evaluated the reliability (internal consistency) of the WHOQOL-BREF questionnaire in patients with cataract. We found that WHOQOL-BREF questionnaire exhibited good internal consistency overall (Cronbach’s alpha of 0.889) and that each of its domains exhibited satisfactory consistency (Cronbach’s alpha >0.7). The reliability analyses of Skevington [26], Nedjat [27], Gholami [28,29], Asnani [6], and Mazaheri [12] indicated that the WHOQOL-BREF scale had an acceptable level of internal consistency (Cronbach’s alpha >0.7); however, Cronbach’s alpha for the social relationship domain was low (<0.7) in those studies. In this study, a positive correlation between all domains of the WHOQOL-BREF was observed. All correlations were found to be statistically significant. In the Gholami [28,29] and Mazaheri [12] studies, a positive correlation between all domains of the WHOQOL-BREF was observed As shown in Table 4, the mean scores of the four domains were significantly different (except for domain 2 and domain 4), with the greatest difference observed between domain 1 and domain 3. Gholami [28,29] and Mazaheri [12] found that the mean scores of four domains were different, with the greatest difference observed between domain 1 and domain 4. In this study, among the four domains of the WHOQOL-BREF, the lowest mean satisfaction rating was found for domain 1 (physical health; mean, 12.19), implying a relatively low activity level in daily life, a greater dependence on medicinal substances and medical aids, insufficient energy and mobility, more pain and discomfort, a lack of sufficient sleep and rest, and a low capacity for work. In contrast, the highest mean score was observed in domain 3 (social relationships, 14.11), implying that the study population had relatively greater levels of satisfaction regarding their personal relationships, sexual activity, and social support. In this study, the highest SD (3.45) was observed in domain 3 (social relationships). In other studies using the WHOQOL-BREF questionnaire, it has also been observed that domain 3 had the highest SD [6,12,27-29]. The fact that the highest SD was observed in domain 3 may be associated with different interpretations of the items used in this domain as well as the small number of items.

In the present study, a multiple linear regression model demonstrated that duration of disease and marital status were significantly associated with the total WHOQOL-BREF score, meaning that single patients and patients with a duration of disease of less than 30 days had a lower quality of life. Age was associated with domain 1 scores, and patients 65 years of age or younger had better physical health. Duration of disease was associated with domain 2, and patients with a duration of disease of less than 30 days had poorer psychological health. Marital status was associated with domain 3, and married patients had a greater quantity of social relationships. Income level was associated with domain 4, and patients with high income reported better environmental health. According to the results of the multiple linear regression model, different variables were associated with each of the four domains of the WHOQOL-BREF.

This study had a number of limitations. First, as this was a cross-sectional study, causality between the compared variables cannot be established. Second, the surveyed population in this study was relatively small.

This study showed the WHOQOL-BREF questionnaire to have good reliability in characterizing the quality of life of patients with cataract. We also found that the surveyed cataract patients had a relatively moderate quality of life. In this study, it was observed that age, marital status, monthly income level, and duration of disease were important variables influencing the quality of life of patients with cataract.

## Figures and Tables

**Table 1.** Characteristics of the study population (n=300)

Characteristics	n	%
Age (yr)		
≤65	108	36
>65	192	64
Sex		
Female	158	52.7
Male	142	47.3
Education level		
Illiterate	196	65.3
Literate	104	34.7
Marital Status		
Unmarried	69	77
Married	231	23
Income level ($/mo)		
≤170	145	59.4
>170	99	40.6
Place of residence		
Urban	163	54.3
Rural	137	45.7
Duration of disease (d)		
≤30	154	51.3
>30	146	48.7

**Table 2.** Responses and missing items for each item (n = 300)

Items (n)	Missing	Mean	SD	Floor	Ceiling
Overall QOL (1)	0 (0)	3.42	0.92	13 (4.3)	20 (6.7)
Overall health (2)	0 (0)	3.48	1.07	22 (7.3)	33 (11.0)
Pain (3)	0 (0)	3.10	1.37	46 (15.3)	66 (22.0)
Dependence on medical aids (4)	0 (0)	3.40	1.14	21 (7.0)	55 (18.3)
Positive feelings (5)	0 (0)	3.11	1.15	35 (11.7)	36 (12.0)
Personal beliefs (6)	0 (0)	3.40	1.00	14 (4.7)	48 (16.0)
Concentration (7)	0 (0)	3.06	1.05	13 (4.3)	32 (10.7)
Security (8)	0 (0)	3.82	0.90	5(1.7)	70 (23.3)
Physical environment (9)	0 (0)	3.76	0.92	3(1.0)	62 (20.7)
Energy (10)	0 (0)	2.79	1.01	24 (8.0)	18 (6.0)
Bodily image (11)	0 (0)	3.67	0.89	5(1.7)	61 (20.3)
Financial support (12)	0 (0)	2.59	0.92	40 (13.3)	7 (2.3)
Accessibility of information (13)	0 (0)	2.71	0.82	20 (6.7)	7 (2.3)
Leisure activities (14)	0 (0)	2.09	0.99	97 (32.3)	5 (1.7)
Mobility (15)	0 (0)	3.13	1.20	33 (11.0)	35 (11.7)
Sleep and rest (16)	0 (0)	3.50	1.12	14 (4.7)	51 (17.0)
Activities of daily living (17)	0 (0)	3.20	1.06	20 (7.6)	22 (7.3)
Work capacity (18)	0 (0)	3.40	0.90	9 (3.0)	16 (5.3)
Self-esteem (19)	0 (0)	3.96	0.90	4(1.3)	91 (30.3)
Personal relationships (20)	0 (0)	3.99	0.97	6 (2.0)	106 (35.3)
Sexual activity (21)	55 (18.3)	3.66	1.03	10 (3.3)	59 (19.7)
Social support (22)	0 (0)	3.60	1.07	16 (5.3)	57 (19.0)
Home environment (23)	0 (0)	3.79	0.85	6 (2.0)	45 (15.0)
Health care (24)	0 (0)	3.64	0.97	10 (3.3)	42 (14.0)
Transport (25)	0 (0)	3.54	0.94	8(2.7)	28 (9.3)
Negative feelings (26)	0 (0)	3.42	1.37	33 (11.0)	100 (33.0)

Values are presented as number (%). SD, standard deviation; QOL, quality of life.

**Table 3.** Correlation coefficients (CC) between the two overall quality of life items (Q1 and Q2) and the four domains (DOM) of the World Health Organization Quality of Life Instrument, Short Form

		Q1	Q2	DOM 1	DOM 2	DOM 3	DOM 4
Q1	CC	1	0.434	0.277	0.237	0.264	0.520
	p-value		< 0.001	< 0.001	<0.001	< 0.001	< 0.001
Q2	CC		1	0.225	0.247	0.235	0.386
	p-value			< 0.001	<0.001	< 0.001	< 0.001
DOM 1	CC			1	0.373	0.313	0.365
	p-value				<0.001	< 0.001	< 0.001
DOM 2	CC				1	0.441	0.480
	p-value					< 0.001	< 0.001
DOM 3	CC					1	0.446
	p-value						< 0.001
DOM 4	CC						1
	p-value						

**Table 4.** Paired t-test for the four domains of the World Health Organization Quality of Life Instrument, Short Form

		Paired differences				t-test	df	p-value (two-tailed)
		Mean	SD	95% CI				
				Lower	Upper			
Pair 1	DOM1-DOM2	-0.90	2.37	-1.16	-0.63	-6.58	299	< 0.001
Pair 2	DOM1-DOM3	-1.82	3.38	-2.21	-1.44	-9.34	299	< 0.001
Pair 3	DOM1-DOM4	-0.92	2.29	-1.18	-0.66	-6.97	299	< 0.001
Pair 4	DOM2-DOM3	-0.92	3.19	-1.29	-0.56	-5.01	299	< 0.001
Pair 5	DOM2-DOM4	-0.02	2.26	-0.28	0.24	-0.15	299	0.88
Pair 6	DOM3-DOM4	0.90	3.15	0.55	1.26	4.97	299	< 0.001

SD, standard deviation; CI, confidence interval; df, degree of freedom; DOM, domain.

**Table 5.** Comparison of the scores in the four domains of the World Health Organization Quality of Life Instrument, Short Form according to independent variables

	Domain 1	Domain 2	Domain 3	Domain 4	Total
Total	12.29±1.91	13.19±2.29	14.11±3.45	13.21±2.13	13.19±1.81
Age (yr)					
≤65	12.84±1.83	13.25±2.30	14.21±3.47	13.54±2.31	13.46±1.87
> 65	11.98±1.88	13.16±2.29	14.06±3.45	13.03±2.01	13.05±1.79
p-value	<0.001	0.73	0.71	0.05	0.07
Sex					
Female	12.49±1.87	13.35±2.22	14.70±3.09	13.01±2	13.39±1.73
Male	12.11±1.92	13.05±2.34	13.59±3.69	13.39±2.24	13.03±1.92
p-value	0.09	0.27	0.005	0.13	0.01
Education level					
Illiterate	12.04±1.87	13.01±2.32	13.74±3.62	12.90±2.02	12.92±1.84
Literate	12.76±1.88	13.53±2.2	14.81±3.02	13.79±2.23	13.72±1.72
p-value	0.002	0.06	0.007	0.001	< 0.001
Marital status					
Married	12.45±1.84	13.27±2.2	14.96±3.23	13.33±2.04	13.51±1.76
Unmarried	11.74±2.05	12.91±2.58	11.28±2.57	12.80±2.41	12.18±1.72
p-value	0.006	0.25	<0.001	0.07	< 0.001
Income level ($/mo)					
≤170	12.14±1.93	13.37±2.42	13.87±3.91	12.79±1.98	13.04±1.90
>170	12.60±1.99	13.66±2.30	14.83±3.21	13.78±2.41	13.71±1.88
p-value	0.07	0.36	0.04	0.001	0.007
Place of residence					
Urban	12.24±1.92	13.21±2.36	14.22±3.39	13.55±2.15	13.31±1.85
Rural	12.35±1.90	13.16±2.21	13.99±3.54	12.81±2.06	13.08±1.82
p-value	0.62	0.84	0.56	0.003	0.28
Duration of disease (d)					
≤30	12.03±1.83	12.63±1.99	13.81±3.08	13.26±2.09	12.93±1.66
> 30	12.56±1.95	13.78±2.44	14.43±3.79	13.16±2.18	13.48±1.97
p-value	0.02	< 0.001	0.12	0.68	0.009

Values are presented as mean±standard deviation.

**Table 6.** Reverse multiple linear regression analyses of factors significantly associated with QOL

QOL domains	Variables	Unstandardized coefficients		Standardized coefficients	t	p-value
		Beta	SE	Beta		
Domain 1	Age	-0.699	0.231	-0.176	-3.028	0.003
Domain 2	Duration of disease	1.151	0.256	0.252	4.491	< 0.001
Domain 3	Marital status	-4.195	0.468	-0.499	-8.968	< 0.001
Domain 4	Income level	0.767	0.306	0.170	2.507	0.01
Total	Marital status	-1.411	0.270	-0.321	-5.224	< 0.001
	Duration of disease	0.550	0.230	0.144	2.388	0.02

QOL, quality of life; SE, standard error.
